# Case study: successful recovery from anorexia nervosa in a 19yr old patient using manualised FBT

**DOI:** 10.1186/2050-2974-2-S1-O7

**Published:** 2014-11-24

**Authors:** Kellie Lavender

**Affiliations:** 1 Regional Eating Disorders Service, Auckland, New Zealand

## 

At the Regional Eating Disorders Service (REDS) in Auckland Family Based Treatment (FBT) is the first line treatment offered to adolescents and their families - and with great success. At REDS we are also offering a choice to individuals over 18 and their families between FBT and individual treatment in an adult part of the service. This presentation describes a successful example of a client case study where FBT was provided with a 19yr old Japanese woman and her family. The case presented with some initial challenges like beginning treatment with a BMI of 14.9, parents needing interpreters and the family living 40km away.

There is no evidence for the effectiveness of FBT for young adults; however in a case series published by Chen., LeGrange et al. (2010) they describe how FBT was used with 4 older clients with 3/4 at follow up being in the normal weight range. The presenter raises the question about whether services should offer FBT as a choice to those over 18 years and living at home and willing to have their family involved.

This presentation will also discuss the question about whether there is a need to consider modifications to FBT with this older age group. A summary of data of FBT cases with young adults at REDS will be provided.

This abstract was presented in the **Treatment in Community and Inpatient Settings** stream of the 2014 ANZAED Conference.

